# Heat shock protein HSPA13 promotes hepatocellular carcinoma progression by stabilizing TANK

**DOI:** 10.1038/s41420-023-01735-0

**Published:** 2023-12-08

**Authors:** Xuesong Cen, Yuyan Lu, Jing Lu, Changhong Luo, Ping Zhan, Yizhe Cheng, Fan Yang, Chengrong Xie, Zhenyu Yin, Fuqiang Wang

**Affiliations:** 1 Department of Hepatobiliary Surgery, Xiamen Key Laboratory of Liver Diseases, Xiamen Hospital of Traditional Chinese Medicine, Beijing University of Chinese Medicine, Xiamen, Fujian Province China; 2 Xiamen Translational Medical Key Laboratory of Digestive System Tumor, Fujian Provincial Key Laboratory of Chronic Liver Disease and Hepatocellular Carcinoma, Zhongshan Hospital of Xiamen University, School of Medicine, Xiamen University, Xiamen, Fujian Province China; 3 Department of Emergency, The Second Hospital of Jiaxing, The Second Affiliated Hospital of Jiaxing University, Jiaxing, Zhejiang Province China

**Keywords:** Oncogenes, Cancer

## Abstract

HSPA13, an important member of the heat shock protein family, plays an essential role in the oncogenesis of many organs, but the mechanism and function in hepatocellular carcinoma (HCC) is still unclear. In the present study, we found that HSPA13 was highly expressed in HCC and predicted poor clinical prognosis. Upregulation of HSPA13 was significantly associated with vascular invasion in HCC patients. Functionally, knockdown experiments demonstrated that HSPA13 promoted HCC proliferation, migration, and invasion. Mechanistic investigation showed that HSPA13 could interact with TANK to inhibit its ubiquitination and degradation. In addition, the expression of HSPA13 and TANK were positively correlated in HCC tissues. To conclude, the present study uncovers the oncogenic function of HSPA13 in the progression of HCC by regulating the stability of TANK. These findings suggest that HSPA13 and TANK may serve as promising targets for the diagnosis and treatment of HCC.

## Introduction

Hepatocellular carcinoma (HCC) is a kind of highly malignant tumor and causes ~830,000 deaths per year, ranked third in tumor mortality [1] Although there are numerous treatments available for HCC, such as radical resection, targeted therapy, transarterial chemoembolization (TACE), the mortality rate of HCC patients is still increasing 2–3% per year, and the situation is still not optimistic [2, 3]. Therefore, it is urgent to actively study the mechanism of HCC oncogenesis to provide clinical direction for treatment.

Heat shock 70 kDa proteins (HSP70s) are essential molecules for the folding and remodeling of cellular proteins and are extremely important in maintaining proteostasis [4, 5]. Previous studies have shown that HSP70s are highly expressed in a variety of cancers, such as breast, pancreatic, colorectal and prostate cancers [6–9]. HSPA13, a member of the HSP70 family, encodes a conserved ATPase structural domain and is capable of interacting with Receptor-interacting protein 1 (RIP1) to regulate TNFα signaling [10]. HSPA13 promotes antibody secretion by plasma cells [11]. In addition, HSPA13 induces prion diseases, implying that HSPA13 may be associated with abnormal protein folding diseases [12]. Despite some studies claiming that HSPA13 is highly expressed in gastric and colon cancer and associated with the recurrence of oral cancer, there is still a lack of research on its role and mechanism in HCC [13–15]. As such, the current understanding of HSPA13 in HCC remains unclear.

TANK (tumor necrosis factor receptor (TNFR) associated factor (TRAF) family member associated NF-κB activator), is a type of TRAF-binding protein. TRAF, a multifunctional family of adaptor proteins, binds to surface receptors and forms signal transduction complexes with various other proteins, playing a crucial role in cell proliferation, differentiation, and stress response [16–18]. TANK participates the development of multiple diseases as a scaffolding protein [19]. In addition, TRAF is also referred to as TARF interacting protein (I-TRAF) and exhibits both stimulatory and inhibitory properties in regulating NF-κB activation [20, 21]. TANK transduces upstream RIG-I-like receptor (RLR) signaling to TANK-binding kinase 1 (TBK1), which induces phosphorylation of interferon regulatory factor 3 (IRF-3) [22–24]. IRF-3 phosphorylation and subsequent dimerization induces IRF-3 nuclear translocation, and IRF-3 subsequently binds interferon-stimulated response elements (ISRE), leading to type I interferon gene expression [25]. Moreover, binding of TANK to TBK1 and TRAF2/6 induces downstream NF-κB-mediated transcription [26, 27]. Prior research has demonstrated that aberrant expression of TANK can trigger activation of the AKT signaling pathway in glioblastoma and is correlated with tumor grade [28, 29]. Nevertheless, the role of TANK in HCC development has not been investigated.

In this study, we examined the expression levels of HSPA13 in HCC tissues and investigated the correlation between HSPA13 expression and clinicopathological features as well as prognosis. Furthermore, we explored the functional role and underlying mechanism of HSPA13 in HCC. Our findings suggest that HSPA13 could serve as a promising therapeutic target for HCC.

## Results

### HSPA13 is highly expressed in HCC tissues and its expression correlates with poor prognosis

**Fig. 1:**
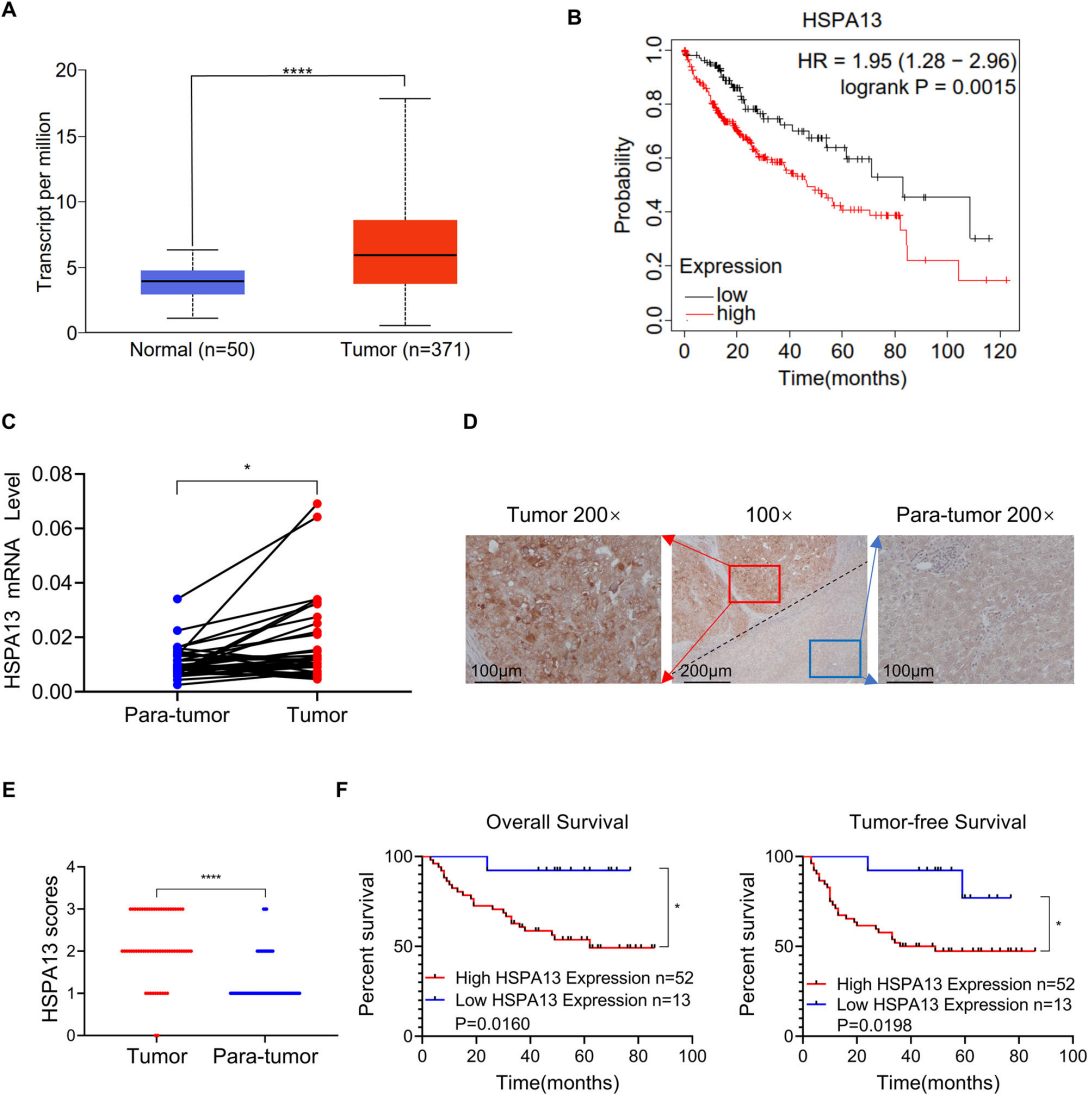
HSPA13 is highly expressed in HCC tissues and its expression correlates with poor prognosis. **A** UALCAN online tool was used to analyze the mRNA expression of HSPA13 in HCC and normal liver tissues from the TCGA database. **B** Kaplan–Meier Plotter tool was used to analyze overall survival rates of HCC patients with high or low HSPA13 expression. **C** The mRNA level of HSPA13 in paired HCC tissues were detected by qPCR. (*n* = 35). **D** Representative IHC staining images of HSPA13 in paired HCC tissues. (*n* = 65). **E** The IHC scores from (**C**) and different expressions of HSPA13 were analyzed by Wilcoxon’s test. (*n* = 65). **F** Kaplan–Meier survival analysis of overall survival and tumor-free survival in HCC patients. (*n* = 65, *p < 0.05, ****p < 0.001).

By analyzing The Cancer Genome Atlas (TCGA) database (http://ualcan.path.uab.edu/ analysis.html), we found that the mRNA expression level of HSPA13 was higher in HCC than in paraneoplastic tissues (Fig. 1A). Besides, through the analysis of Kaplan–Meier Plotter online tool, the patients with high expression of HSPA13 had a poorer prognosis compared to those with low HSPA13 expression (Fig. 1B). Meanwhile, we examined HSPA13 mRNA levels in 35 pairs of HCC and paraneoplastic tissues and found that HSPA13 expression was higher in 68.7% (24/35) of HCC tissues than in para-cancerous tissues (Fig. 1C). Afterwards, we conducted an investigation into the levels of the HSPA13 protein in HCC and paraneoplastic tissues of 65 patients using immunohistochemical (IHC) staining. The results revealed that HSPA13 was predominantly expressed in the cytoplasm and in 80% (52/65) of the patients, HSPA13 expression was higher in HCC tissues than in para-tumor tissues (Fig. 1D, E). Furthermore, survival analysis demonstrated that high expression of HSPA13 was inversely correlated with overall and tumor-free survival (Fig. 1F). Therefore, HSPA13 could serve as a significant prognostic indicator for HCC patients.

In order to investigate the clinical significance of HSPA13 in more depth, we analyzed the relationship between pathological factors and HSPA13 expression. As shown in Table 1, high expression of HSPA13 was significantly associated with vascular invasion (*P* = 0.03). However, the association with other factors was not obvious. Taken together, HSPA13 may facilitate the development of HCC as a proto-oncogenic molecule.

**Table 1:** Association between HSPA13 expression and clinicopathologic characteristics in HCC.

Clinicopathological characteristics	HSPA13 Expression		*p*
	High	Low	
Age			
≥60	22	4	0.45
<60	30	9	
Gender			
Female	12	1	0.44
Male	40	12	
Tumor size			
≤5 cm	12	4	0.72
>5 cm	40	9	
Differentiation level			
High/Middle	45	13	0.33
Low	7	0	
AFP (ug/L)^a^			
<200	30	9	0.54
≥200	21	4	
Vascular invasion			
Without	20	10	0.03*
With	32	3	
HBV DNA^b^			
<1000	13	4	0.08
≥1000	22	9	
Satellite foci			
Without	38	9	0.74
With	14	4	
Liver cirrhosis^c^			
Without	9	1	0.67
With	40	11	

*AFP* Alpha-fetoprotein, *HBV* Hepatitis B virus.

*p < 0.05, **p < 0.01.

^a^1 missing data points.

^b^17 missing data points.

^c^4 missing data points.

### HSPA13 promotes the proliferation, migration, and invasion of HCC cells

**Fig. 2:**
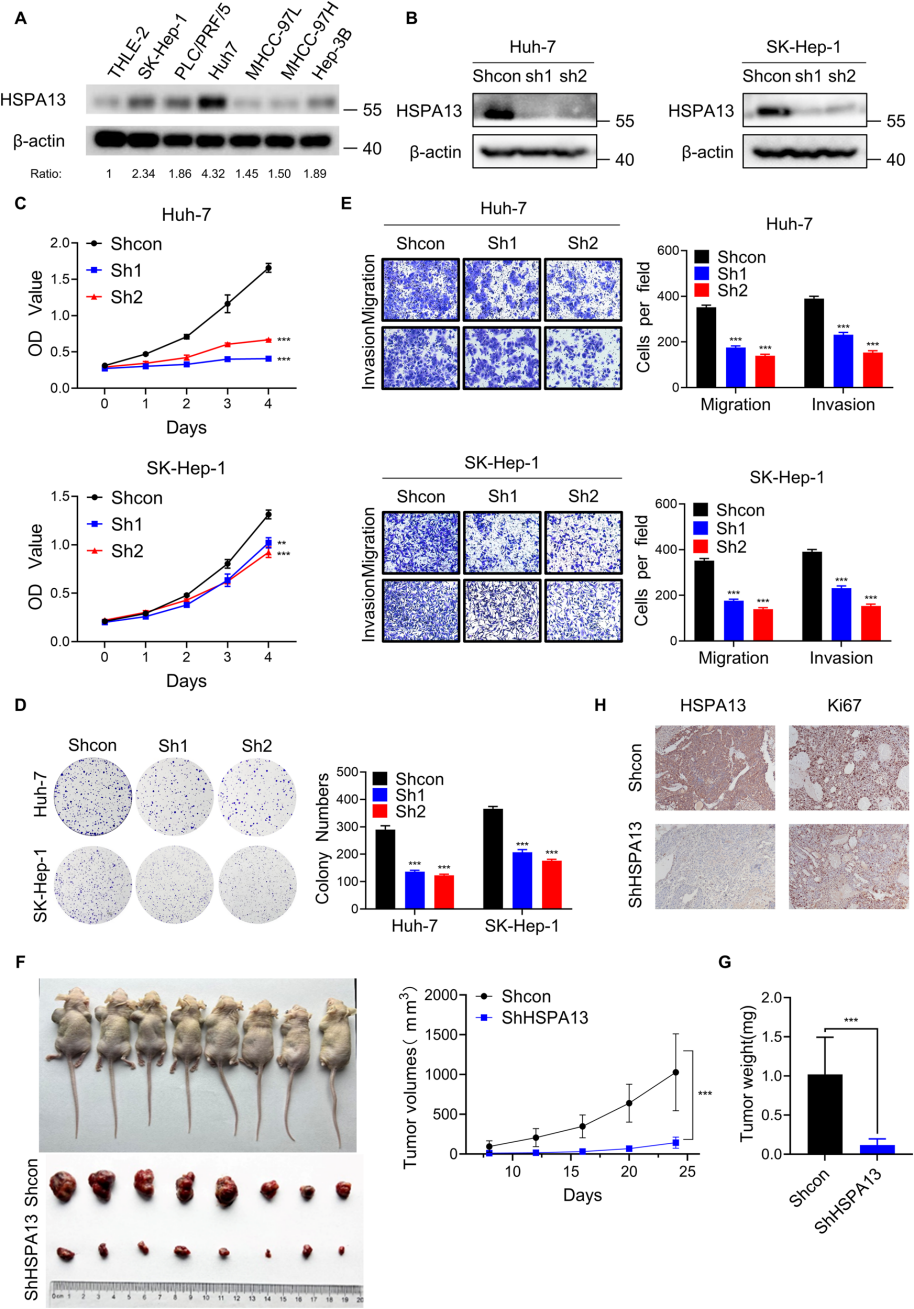
HSPA13 promotes the proliferation, migration and invasion of HCC cells. **A** HSPA13 protein levels were determined in HCC cell lines and a normal liver cell line THLE-2 by western blot. **B** Huh-7 and SK-Hep-1 cells were infected with lentiviral particles expressing HSPA13 shRNAs and the knockdown efficacy was validated by western blot. **C** CCK-8 assay was performed to detect the proliferative capacity of control and HSPA13 silenced HCC cells. **D** Colony formation assay was used to verify the proliferative capacity of control and shHSPA13 HCC cells. **E** The representative images of migration and invasion for control and shHSPA13 cells. Five random fields were selected for cell counting under 100× microscopes. **F** Control and shHSPA13 HCC cells were injected on the left and right sides of nude mice, respectively. The images and growth curve of xenograft tumors were shown. **G** Tumor weight was used to measure the effects of HSPA13 on proliferation in vivo. **H** The efficiency of knocking down HSPA13 and its effect on Ki67 were confirmed by IHC staining. (**p < 0.01, ***p < 0.001).

Based on our observation of high expression of HSPA13 in HCC tissues, the function of HSPA13 in HCC cells was explored. The expression of HSPA13 in HCC cell lines and a normal liver cell line THLE-2 cells was firstly examined. HCC cell lines expressed higher HSPA13 levels than THLE-2 cells did (Fig. 2A). Considering the endogenous expression levels and knockdown efficiency, we selected Huh-7 and SK-Hep-1 cells for knocking down experiments (Fig. 2B). CCK-8 assay and colony formation assay revealed that knockdown of HSPA13 inhibited the proliferation and survival of HCC cells (Fig. 2C, D, Supplementary Fig. 1). Then, transwell assay verified that knockdown of HSPA13 significantly inhibited the migration and invasion of HCC cells (Fig. 2E).

To detect the role of HSPA13 in vivo, control and HSPA13 knockdown Huh-7 cells were injected subcutaneously on the left and right side of nude mice, respectively. After 24 days, we found that the final isolated tumors showed that HSPA13-deleted tumors were significantly smaller in volume and weight than the control group (Fig. 2F, G). The knockdown efficacy was also verified by IHC staining (Fig. 2H). Ki67 is a nuclear protein expressed throughout the cell cycle except the G0 phase [30]. This protein is mainly expressed in proliferating cells and considered to be the marker of cell proliferation [31]. By IHC staining, we observed that Ki67 expression was significantly downregulated in xenograft tumors with knockdown of HSPA13 (Fig. 2H). Thus, these results demonstrate that HSPA13 promotes the growth of HCC.

### HSPA13 inhibits the ubiquitination and degradation of TANK in HCC cells

**Fig. 3:**
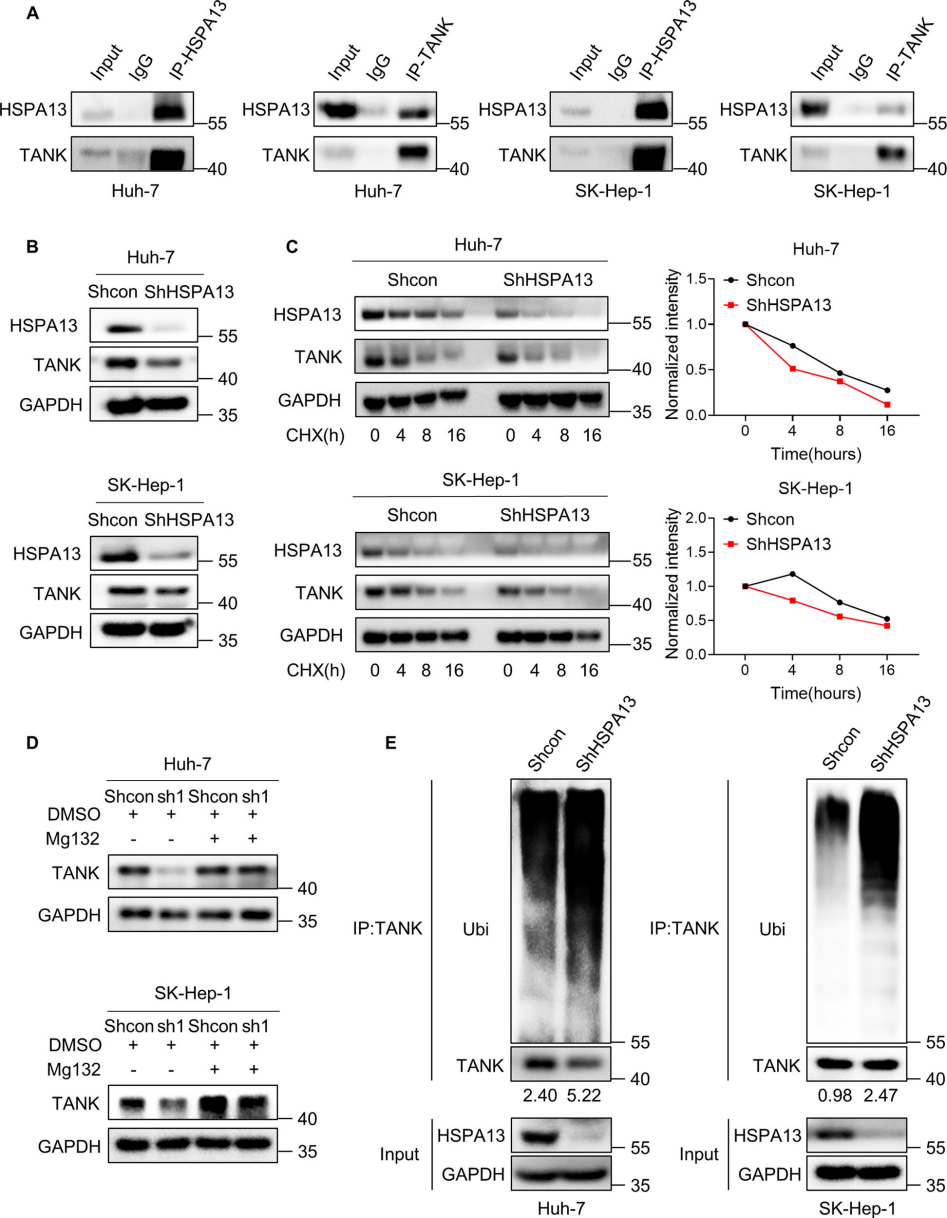
HSPA13 inhibits the ubiquitinated degradation of TANK. **A** Co-immunoprecipitation of endogenous HSPA13 and TANK in Huh-7 and SK-Hep-1 cells. **B** Detection of protein levels of TANK by western blot after knocking down HSPA13. **C** The control and knockdown HSPA13 cells were treated with CHX (100 μg/ml) for the indicated time gradients. Western blot was used to detect levels of protein (left panel). TANK protein levels were quantified by ImageJ (right panel). **D** The control and HSPA13 knockdown Huh-7 and SK-Hep-1 cells were pre-treated with DMSO and Mg132 (25 µM) for 10 h. Then protein levels of HSPA13, TANK, and GAPDH were measured via western blot. **E** Control and shHSPA13 Huh-7 and SK-Hep-1 cells was incubated with Mg132 (25 µM) for 10 h. And then the ubiquitination levels of TANK were detected by western blot.

To further investigate the mechanisms by which HSPA13 regulates HCC progression, the HSPA13-interacting proteins were predicted by HitPredict tool. We selected 15 proteins with the highest scores and reviewed literatures to screen the proteins that may closely associated with HSPA13. Eventually, HYOU1, FBX6, and TANK (TRAF family member-associated NF-kappa-B activator) were found to be potential interacting proteins of HSPA13 (Supplementary Fig. 2A). However, through co-IP assay, it was verified that only TANK could bind to HSPA13 (Fig. 3A, Supplementary Fig. 2B and C). It has been reported that TRAF family proteins display abnormal expression patterns in a diverse range of cancers and are linked to proliferation and differentiation processes [32, 33]. To investigate the regulatory role of HSPA13 on TANK, we conducted a western blot analysis to assess the protein expression level of TANK. Our results revealed that the protein level of TANK was significantly reduced upon knocking down HSPA13 in Huh-7 and SK-Hep-1 cells (Fig. 3B).

Next, we conducted a CHX pulse-chase assay to investigate the impact of HSPA13 knockdown on endogenous TANK protein stability. The results revealed that HSPA13 knockdown expedited the degradation of TANK protein in SK-Hep-1 and Huh-7 cells (Fig. 3C). To elucidate the mechanism of TANK degradation, HCC cells were treated with the proteasome inhibitor MG132. The decrease of TANK mediated by HSPA13 downregulation could be rescued by MG132 treatment (Fig. 3D). Furthermore, we employed the ubiquitination assay to determine whether HSPA13 regulates the ubiquitination level of TANK. Our findings indicated that HSPA13 knockdown significantly increased the ubiquitination level of TANK (Fig. 3E). In summary, these results demonstrate that HSPA13 knockdown accelerates TANK degradation through the ubiquitin-proteasome pathway.

### Downregulation of TANK suppresses HCC cells proliferation, migration, and invasion

**Fig. 4:**
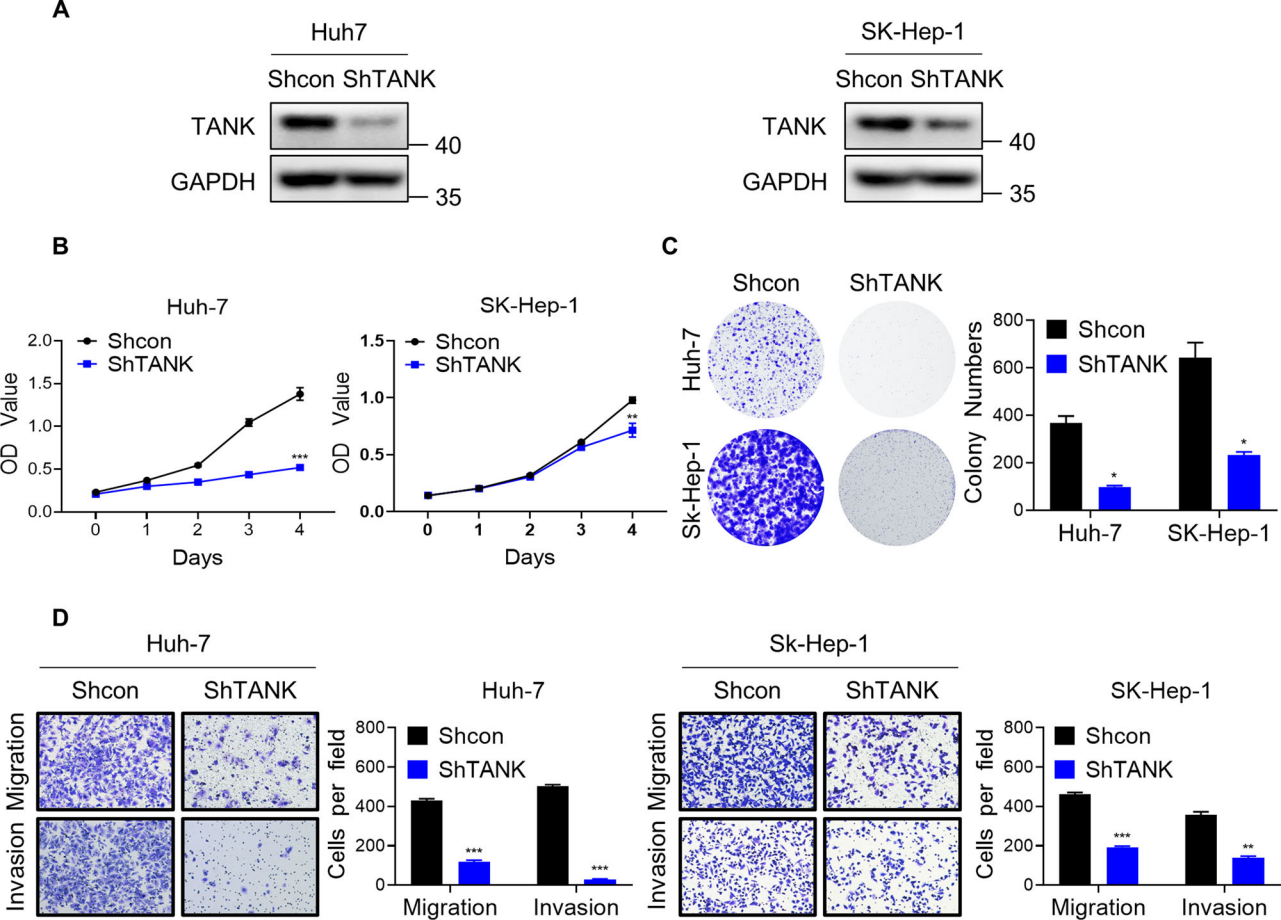
Downregulation of TANK inhibits the proliferation, migration, and invasion of HCC cells. **A** Phosphorylation levels of AKT were measured via western blot after TANK was knocked down. **B** The proliferative capacity of control and shTANK HCC cells were examined by CCK-8 assays. **C** Colony formation assays were used to detect the clonogenic ability of control and TANK-deleted HCC cells. **D** The representative images of migration and invasion for control and shTANK HCC cells. (*p < 0.05, **p < 0.01, ***p < 0.001).

Based on the finding that HSPA13 regulates TANK, we next detected the function of TANK in HCC cells. First, we examined the knockdown efficacy of TANK at the protein level (Fig. 4A). Meanwhile, CCK-8 and clone formation assays implied that knockdown of TANK significantly suppressed the proliferation of HCC cells (Fig. 4B, C). Furthermore, transwell assays revealed a significant reduction in the migration and invasion ability of HCC cells following TANK knockdown (Fig. 4D). These results strongly suggest that TANK functions as a proto-oncogene in HCC.

### HSPA13 exerts oncogenic effects partially through TANK

**Fig. 5:**
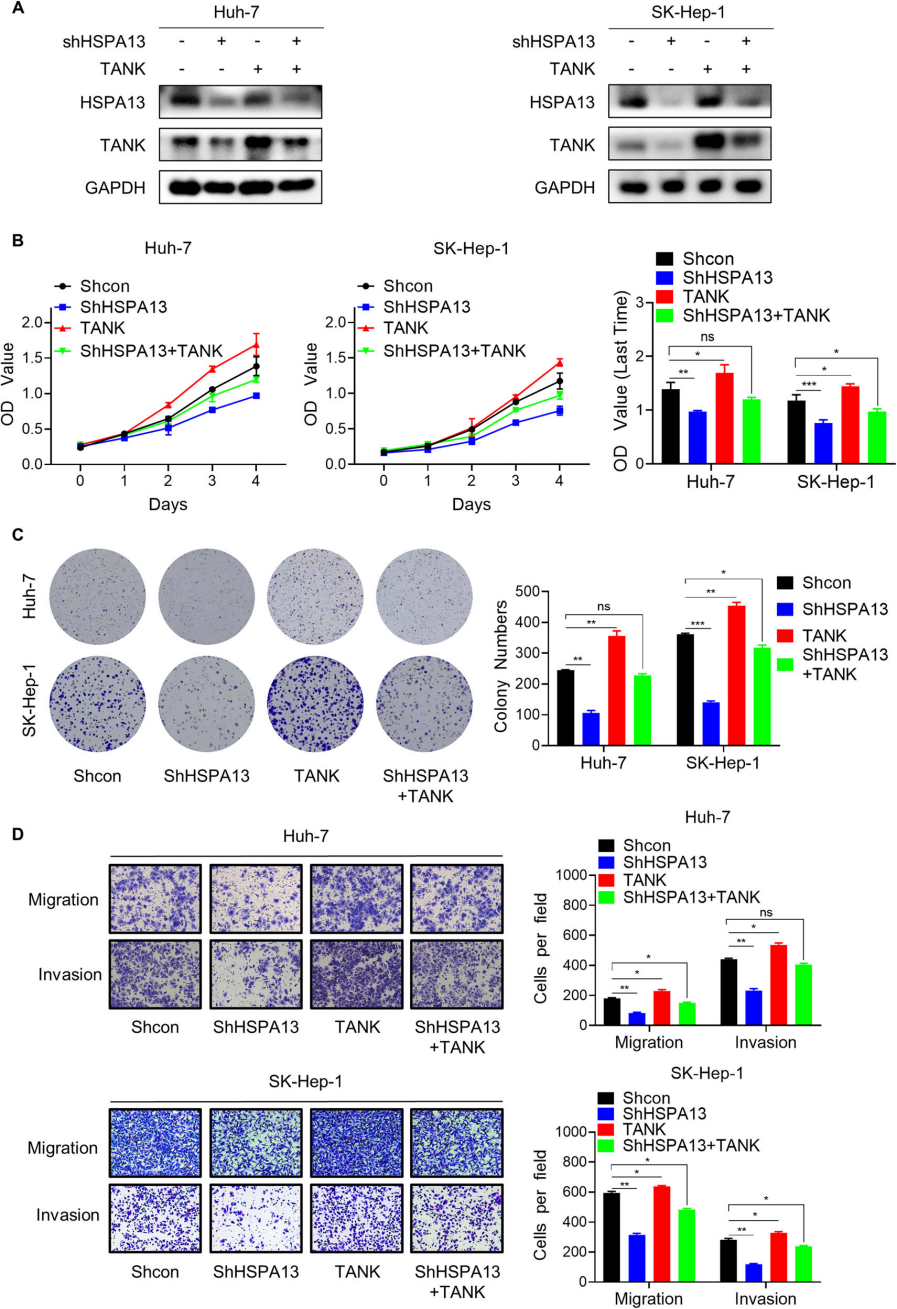
HSPA13 exerts oncogenic effects partially through TANK. **A** The effect of reintroducing TANK into HCC cells with stable shHSPA13 was assessed by western blot. **B** TANK partially abolished the proliferation inhibition induced by HSPA13 knockdown in Huh-7 and SK-Hep-1 cells as confirmed by CCK-8 assay. **C** TANK partially abolished the colony formation capability inhibition induced by HSPA13 knockdown in Huh-7 and SK-Hep-1 cells as confirmed by colony formation assay. **D** TANK partially abolished the migration and invasion inhibition induced by HSPA13 knockdown in Huh-7 and SK-Hep-1 cells as confirmed by transwell assay. (*p < 0.05, **p < 0.01, ***p < 0.001).

Expanding upon our above findings, we delved deeper into the pivotal role of the HSPA13-TANK axis in HCC cells. To this end, we conducted a rescue experiment wherein HSPA13 was knocked down following TANK overexpression in HCC cells (Fig. 5A). Our data further revealed that overexpression of TANK partially reversed the decrease in proliferation, migration, and invasion of HCC cells caused by HSPA13 downregulation (Fig. 5B–D). These findings unequivocally demonstrate that HSPA13 regulates the progression of HCC, in part, through its interaction with TANK.

### HSPA13 positively correlates with TANK expression in HCC tissues

**Fig. 6:**
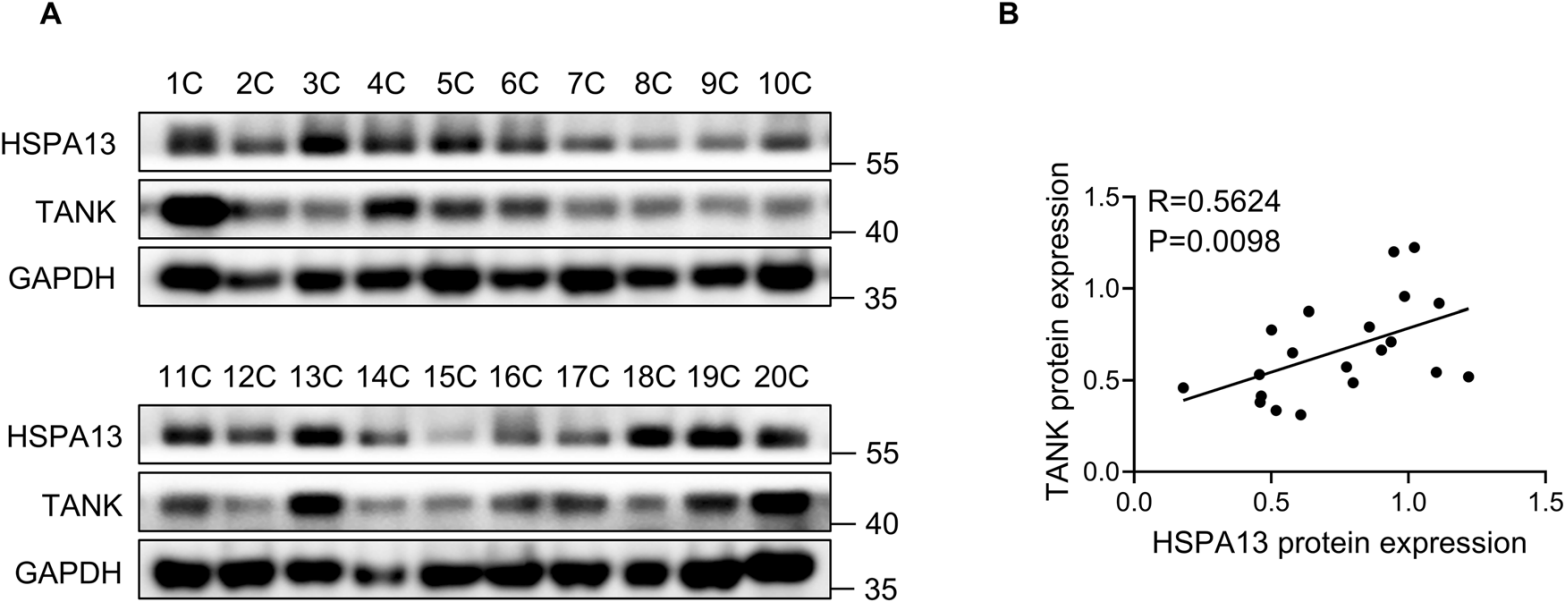
HSPA13 positively correlates with TANK protein expression in HCC tissues. **A** The protein expressions of HSPA13 and TANK in HCC tissues were detected by western blot. **B** Spearman’s test was performed to analyze the correlation between HSPA13 and TANK (*R* = 0.5624, *P* = 0.0098).

To evaluate the pathological significance of HSPA13 and TANK protein expression levels, we proceeded to analyze the protein expression of these two factors in 20 HCC tissues through western blotting (Fig. 6A). Pearson’s correlation analysis demonstrated a positive correlation between the protein levels of HSPA13 and TANK in HCC tissues (Fig. 6B). In summary, our findings suggest that the upregulation of HSPA13 and TANK may serve as promising prognostic indicators for patients with HCC.

## Discussion

The current study demonstrated that HSPA13 was involved in the growth and metastasis of HCC and may serve as an indicator of poor prognosis. HCC patients with high expression of HSPA13 have shorter overall survival and tumor-free survival rates. Clinically, high expression of HSPA13 is associated with vascular invasion in patients with HCC. The invasion and metastasis of cancer are critical factors that contribute to poor clinical outcomes. Epithelial-mesenchymal transition (EMT) is a crucial process that impacts tumor metastasis. Previous studies have indicated that HSP70s in colon cancer can enhance EMT and affect key indicators such as slug, snail, E-cadherin, smad2/3, and twist [34, 35]. In lung cancer, high expression of HSP70s has been found to promote SUMO modification of hypoxia-inducible factors-1α (HIF-1α) and consequently promote EMT in tumor cells. Moreover, the expression of HSP70s has been found to be negatively correlated with E-cadherin [36]. Based on these findings, we speculate that HSPA13 may promote tumorigenesis by affecting key molecules of EMT, such as decreasing E-cadherin expression and promoting vimentin expression, which in turn promotes EMT and the metastasis of tumor cells.

Previous studies have reported upregulation of HSPA13 in HCC, but the underlying mechanism and phenotype have not been thoroughly investigated [4, 14]. Our study aimed to fill this gap and found that HSPA13 promotes the proliferation, migration, and invasion of HCC cells in vitro, and facilitates tumor growth in vivo. HSPA13 has been recognized as a proto-oncogene in various cancers, including colon cancer, breast cancer, and cutaneous melanoma. For example, in colon cancer, knockdown of HSPA13 accelerates apoptosis and necrosis, facilitating ubiquitination of RIP1 in response to TNFα induction [10, 14]. In addition to this, HSPA13 promotes invasion and metastasis of breast cancer cells by regulating angiogenesis and nutrient supply [37]. Moreover, HSPA13 is a risk gene in cutaneous melanoma and is associated with poor patient prognosis [38]. Collectively, these studies, along with our findings, support the notion that HSPA13 expression positively regulates tumorigenesis. In addition, A previous study has reported that TANK could be phosphorylated by IKKbeta in response to TNFα stimulation, leading to the activation of NF-κB [39]. As a scaffold protein, TANK participates in the assembly of the TBK1-IKKepsilon complex and is subject to lipopolysaccharide (LPS) and TBK1-IKKepsilon-mediated Lys(63)-linked polyubiquitination [40]. TANK has also been reported to be aberrantly expressed in cervical cancer and is associated with apoptosis in osteosarcoma [41, 42]. Our research revealed that TANK promotes the malignant behavior and progression of HCC, which is consistent with previous studies. In summary, TANK is also a pro-oncogene in HCC.

HSP70s play a dual role in protein degradation, acting as both inhibitors and promoters. They facilitate the degradation of misfolded proteins while also stabilizing certain proteins, such as tumor necrosis factor receptor-associated factor 6 (TRAF6) [43, 44]. Previous studies have shown that HSP70s promote the progression of gastric cancer by stabilizing S phase kinase-associated protein 2 (SKP2) [45]. Similarly, in gliomas, overexpression of HSP70s greatly maintains the stability of activating transcription factor 5 (ATF5), preventing its degradation through protease-dependent and caspase-dependent processes. While we discovered that TANK could interact with HSPA13, the relationship between HSPA13 and TANK and their mechanism in HCC has not been reported. Our research revealed that knockdown of HSPA13 accelerated the degradation of TANK. Furthermore, deletion of HSPA13 significantly increased the ubiquitination of TANK, suggesting a potential mechanism underlying the relationship between HSPA13 and TANK in HCC. Interestingly, a recent study showed that HBP21, a member of HSP70s, could promote interferon regulatory factor 3 (IRF3) phosphorylation and facilitate the formation of the TBK1-IRF3 complex [46]. Meanwhile, TANK and TBK1 can stabilize each other in malignant glioma, with TBK1 being crucial to TANK’s function [28]. Therefore, we suspect that HSPA13 may have a similar function and stabilize TANK by regulating TBK1.

In summary, our study reveals that the overexpression of HSPA13 is a predictor of unfavorable prognosis in patients with HCC. Furthermore, the stabilization of TANK by HSPA13 and the activation of AKT contribute to the growth and metastasis of HCC. These findings highlight promising targets for the treatment of HCC and offer new avenues for clinical therapy.

## Materials and methods

### Specimen collection

65 pairs of human HCC tissues and paracancerous tissues as well as pathological and follow-up information were provided by Zhongshan Hospital of Xiamen University. All cases were collected from 2016 to 2020. The study was approved by the Ethics Committee of Zhongshan Hospital, Xiamen University, and all patients signed an informed consent form.

### Immunohistochemical staining (IHC)

Specimens were formalin-fixed and embedded in paraffin, followed by dewaxing of 4 μm sections. Rehydration was performed in the order of 100–70% alcohol. Antigen repair was performed with citric acid repair solution at high temperature and pressure, blocked with hydrogen peroxide and then closed for one hour, followed by overnight incubation with anti-HSPA13 antibody (1:2000, 12667-2-AP, Proteintech). Secondary antibody incubation with goat anti-rabbit IgG at room temperature for 30 minutes. Finally, they were stained with diaminobenzidine (DAB, Maixin Biotechnology, Fuzhou, China) and hematoxylin (Maixin Biotechnology). All sections were reviewed by two pathologists. We counted 100 cells randomly at 200 × microscopic fields and classified them into four groups according to the percentage of positive staining cells in HCC tissues as follows: 0 = negative; 1 = 1–35% (weakly stained); 2 = 36–70% (moderately stained); 3å 70% (strongly stained). The score obtained was considered positive if it was greater than 1.

### Cell culture

Human HCC cell lines, SK-Hep-1, Huh-7, PLC/PRF/5, MHCC-97L, MHCC-7H, and Hep-3B, are purchased from Cellcook Biotechnology Company (Guangzhou, China) and cultured at 37 °C in 5% CO_2_ with high glucose Dulbecco’s modified Eagle medium (DMEM; Gibco) containing 10% fetal bovine serum (FBS; Vivacell). A normal liver cell line THLE-2 was obtained from MeisenCTCC Company (Hangzhou, China) and cultured with Bronchial Epithelial Cell Growth Medium B (BEGM) containing 10% FBS (Vivacell).

### Construction of stable cells

For stable knockdown, short-hairpin RNA (shRNA) sequences were inserted into the pLV-shRNA-puro plasmid. The target sequences of shRNA were shown as follows: ShHSPA13-1: 5’-GTGGGATATGAAAGCGTAG-3’; ShHSPA13-2: 5’-GCCGTCAAGTCATTCAAGAG-3’; ShTANK: 5’-GCCTATACAGTGTACGGATAA-3’). Lentiviral particles were produced in HEK293T cells according to the standard protocol. After 48 h, Lentiviruses were collected and used to infect HCC cells with 8 μg/L of polybrene (Solarbio, Beijing, China). The stable cells were screened by exposing them to 1 μg/ml of puromycin (Solarbio, Beijing, China) for a duration of 7 days.

### Overexpression of TANK

The vector and TANK overexpression plasmid were purchased from the Public Protein/Plasmid Repository (PPL, Nanjing; Jiangsu Province, China). Plasmids were transfected into the cells by using Lipofectamine™ 3000 (Thermo Fisher Scientific) according to the instructions. 24 h later, cells were collected for further experiments.

### Western blot

The collected cells were lysed with RIPA reagent (Beyotime; Beijing, China) and centrifuged for 10 min, then the protein solution was collected and measured by BCA protein assay kit (Thermo Fisher Scientific). For western blotting, protein samples were isolated by sodium dodecyl sulfate-polyacrylamide gel (SDS-PAGE) and transferred onto polyvinylidene difluoride (PVDF) membranes (Millipore), followed by 1 h closure with skim milk and overnight incubation with anti-HSPA13 antibody (12667-2-AP, Proteintech), anti-β-actin (3700, Cell Signaling) or anti-GAPDH antibody (Proteintech, 60004-1-Ig) at 4 °C. The following day, the membranes were washed with TBST and incubated with secondary antibody at room temperature for 1 h. Finally, the protein bands were visualized using an ECL detection system (Millipore). The original images of western blots were shown in Supplementary Fig. 3.

### Co-immunoprecipitation (co-IP)

Proteins from cells were extracted with Cell lysis buffer for Western and IP (Beyotime, Beijing), and then incubated with anti-HSPA13 antibody (12667-2-AP, Proteintech) or anti-TANK (1:50, 2141S, Cell Signaling Technology) antibody or anti-IgG antibody (2729, Cell Signaling Technology) with Dynabeads Protein G (10001D, Invitrogen) for immunoprecipitation overnight at 4 °C. Washing the beads for three times with lysates, then heating at 100 °C for ten minutes. The immunoprecipitated proteins were harvested for western blot.

### Transwell assay

Cells were resuspended with serum-free medium, and a certain amount of cells were placed in the upper chamber (8-μm pore size, Corning Incorporated; New York, NY, USA) and DMEM medium containing 10% FBS was added to the lower layer. The duration of migration and invasion experiments was 24 and 36 h, respectively. Subsequently, they were fixed with 4% paraformaldehyde, stained with crystalline violet, and microscopically selected for photographic counting in five consecutive fields of view.

### Cell counting kit-8 (CCK-8) and colony formation assay

For the CCK-8 assay, 2000 HCC cells were seeded into 96-well plates, and the proliferation of cells was detected by CCK-8 kit (Dojindo; Beijing, China) at 0, 24, 28, 72, and 96 h, respectively. For the colony formation assay, 2000 cells were cultured in six-well plates for one week, after which they were fixed with 4% paraformaldehyde for half an hour, stained with crystal violet and counted.

### Protein half-life assay

Stable cells were treated by cycloheximide (CHX) at a concentration of 100 μg/ml. Then, the cells were harvested at 0, 4, 8, 16 h respectively and analyzed by western blotting.

### Ubiquitination assay

HCC cells were treated with 25 µM of Mg132 (Selleck) for 10 h. Then the collected cells were lysed with RIPA reagent. The obtained protein solutions were incubated overnight with anti-TANK antibody (2141S, Cell Signaling Technology) and Dynabeads Protein G (10001D, Invitrogen) for immunoprecipitation. The enriched TANK proteins were finally detected by immunoblotting with antibody against ubiquitin (3936, Cell Signaling Technology).

### Animal study

A xenograft mouse model was established using 4-week-old male nude mice. The control and HSPA13 knockdown Huh-7 cells were injected into the left and right sides of the mice, respectively. Isolated tumors were weighed out, then fixed in formalin and embedded in paraffin. The 4 μm sections were cut and immunohistochemically stained with anti-HSPA13 antibody (12667-2-AP, Proteintech) and anti-Ki67 antibody (27309-1-AP, Proteintech).

### Quantitative real-time PCR (qPCR)

Total RNA was separated from cells or tissues by Trizol reagent (Ambion, 15596018). Complementary DNA (cDNA) was acquired from reverse transcription of 1 μg of total RNA using HiScript® III All-in-one RT SuperMix perfect for qPCR (Vazyme, R333-01) based on the instructions. qPCR experiments were performed with Taq Pro Universal SYBR qPCR Mater Mix (Vazyme, Q712-02). The primers used for qPCR were listed as follow: HSPA13-forward: 5’-GTCTTCCACGTCTTGGTGATAG-3’; HSPA13-reverse: 5’-CAGACATTGCTCGGGTTAGAA-3’; GAPDH-forward: 5’-GGTGTGAACCATGAGAAGTATGA-3’; GAPDH-reverse: 5’-GAGTCCTTCCACGATACCAAAG-3’.

### Statistical analysis

Graphpad Prism8 and SPSS 25.0 (IBM Corporation, New York, USA) were used for all experimental data analysis. Differences between samples were tested by Student t-test. The relationship between HSPA13 and immunohistochemistry-related pathological factors were analyzed using Chi-square test. Kaplan–Meier was performed to examine whether the survival curves were significant. The correlation between HSPA13 and TANK was detected by Spearman’s correlation analysis. Differences were considered statistically significant when *p* < 0.05.

### Supplementary information

Supplementary figure legends

Supplementary figure 1

Supplementary figure 2

Supplementary figure 3

## Data Availability

The data support the findings of this study are available from the corresponding authors upon reasonable request.

## References

[1] Sung H, Ferlay J, Siegel RL, Laversanne M, Soerjomataram I, Jemal A (2021). Global Cancer Statistics 2020: GLOBOCAN Estimates of Incidence and Mortality Worldwide for 36 Cancers in 185 Countries. CA Cancer J Clin.

[2] Wang F, Zhao W, Gao Y, Zhou J, Li H, Zhang G (2019). CDK5-mediated phosphorylation and stabilization of TPX2 promotes hepatocellular tumorigenesis. J Exp Clin Cancer Res.

[3] Wang W, Wei C (2020). Advances in the early diagnosis of hepatocellular carcinoma. Genes Dis.

[4] Wang B, Lan T, Xiao H, Chen ZH, Wei C, Chen LF (2021). The expression profiles and prognostic values of HSP70s in hepatocellular carcinoma. Cancer Cell Int.

[5] Rosenzweig R, Nillegoda NB, Mayer MP, Bukau B (2019). The Hsp70 chaperone network. Nat Rev Mol Cell Biol.

[6] Miao W, Fan M, Huang M, Li JJ, Wang Y (2019). Targeted profiling of heat shock proteome in radioresistant breast cancer cells. Chem Res Toxicol.

[7] Giri B, Sethi V, Modi S, Garg B, Banerjee S, Saluja A (2017). Heat shock protein 70 in pancreatic diseases: friend or foe. J Surg Oncol.

[8] Moradi-Marjaneh R, Paseban M, Moradi Marjaneh M (2019). Hsp70 inhibitors: Implications for the treatment of colorectal cancer. IUBMB Life.

[9] Kumar S, Gurshaney S, Adagunodo Y, Gage E, Qadri S, Sharma M (2018). Hsp70 and gama-Semino protein as possible prognostic marker of prostate cancer. Front Biosci (Landmark Ed).

[10] Gao C, Deng J, Zhang H, Li X, Gu S, Zheng M (2021). HSPA13 facilitates NF-κB-mediated transcription and attenuates cell death responses in TNFα signaling. Sci Adv.

[11] He Y, Xu R, Zhai B, Fang Y, Hou C, Xing C (2020). Hspa13 promotes plasma cell production and antibody secretion. Front Immunol.

[12] Grizenkova J, Akhtar S, Hummerich H, Tomlinson A, Asante EA, Wenborn A (2012). Overexpression of the Hspa13 (Stch) gene reduces prion disease incubation time in mice. Proc Natl Acad Sci USA.

[13] Aoki M, Yamamoto K, Ohyama S, Yamamura Y, Takenoshita S, Sugano K (2005). A genetic variant in the gene encoding the stress70 protein chaperone family member STCH is associated with gastric cancer in the Japanese population. Biochem Biophys Res Commun.

[14] Guan Y, Zhu X, Liang J, Wei M, Huang S, Pan X (2021). Upregulation of HSPA1A/HSPA1B/HSPA7 and downregulation of HSPA9 were related to poor survival in colon cancer. Front Oncol.

[15] Chung CM, Lee CH, Chen MK, Lee KW, Lan CE, Kwan AL (2017). Combined genetic biomarkers and betel quid chewing for identifying high-risk group for oral cancer occurrence. Cancer Prev Res.

[16] Arch RH, Gedrich RW, Thompson CB (1998). Tumor necrosis factor receptor-associated factors (TRAFs)–a family of adapter proteins that regulates life and death. Genes Dev.

[17] Bradley JR, Pober JS (2001). Tumor necrosis factor receptor-associated factors (TRAFs). Oncogene.

[18] Chung JY, Park YC, Ye H, Wu H (2002). All TRAFs are not created equal: common and distinct molecular mechanisms of TRAF-mediated signal transduction. J Cell Sci.

[19] Pang Y, Ma M, Wang D, Li X, Jiang L (2021). TANK promotes pressure overload induced cardiac hypertrophy via activating AKT signaling pathway. Front Cardiovasc Med.

[20] Rothe M, Xiong J, Shu HB, Williamson K, Goddard A, Goeddel DV (1996). I-TRAF is a novel TRAF-interacting protein that regulates TRAF-mediated signal transduction. Proc Natl Acad Sci USA.

[21] Cheng G, Baltimore D (1996). TANK, a co-inducer with TRAF2 of TNF- and CD 40L-mediated NF-kappaB activation. Genes Dev.

[22] Fitzgerald KA, McWhirter SM, Faia KL, Rowe DC, Latz E, Golenbock DT (2003). IKKepsilon and TBK1 are essential components of the IRF3 signaling pathway. Nat Immunol.

[23] Liu S, Cai X, Wu J, Cong Q, Chen X, Li T (2015). Phosphorylation of innate immune adaptor proteins MAVS, STING, and TRIF induces IRF3 activation. Science.

[24] Zhao B, Shu C, Gao X, Sankaran B, Du F, Shelton CL (2016). Structural basis for concerted recruitment and activation of IRF-3 by innate immune adaptor proteins. Proc Natl Acad Sci USA.

[25] Zhao B, Du F, Xu P, Shu C, Sankaran B, Bell SL (2019). A conserved PLPLRT/SD motif of STING mediates the recruitment and activation of TBK1. Nature.

[26] Pomerantz JL, Baltimore D (1999). NF-kappaB activation by a signaling complex containing TRAF2, TANK and TBK1, a novel IKK-related kinase. EMBO J.

[27] Abe T, Barber GN (2014). Cytosolic-DNA-mediated, STING-dependent proinflammatory gene induction necessitates canonical NF-κB activation through TBK1. J Virol.

[28] Stellzig J, Chariot A, Shostak K, Ismail Goktuna S, Renner F, Acker T (2013). Deregulated expression of TANK in glioblastomas triggers pro-tumorigenic ERK1/2 and AKT signaling pathways. Oncogenesis.

[29] Conti A, Ageunnouz M, La Torre D, Cardali S, Angileri FF, Buemi C (2005). Expression of the tumor necrosis factor receptor-associated factors 1 and 2 and regulation of the nuclear factor-kappaB antiapoptotic activity in human gliomas. J Neurosurg.

[30] Miller I, Min M, Yang C, Tian C, Gookin S, Carter D (2018). Ki67 is a graded rather than a binary marker of proliferation versus quiescence. Cell Rep..

[31] Lashen A, Toss MS, Green AR, Mongan NP, Rakha E (2022). Ki67 assessment in invasive luminal breast cancer: a comparative study between different scoring methods. Histopathology.

[32] Zhu S, Jin J, Gokhale S, Lu AM, Shan H, Feng J (2018). Genetic alterations of TRAF PRoteins in Human Cancers. Front Immunol.

[33] Chapard C, Hohl D, Huber M (2012). The role of the TRAF-interacting protein in proliferation and differentiation. Exp Dermatol.

[34] Nikotina AD, Vladimirova SA, Komarova EY, Alexeev D, Efremov S, Leonova E, et al. Prevention of High Glucose-Mediated EMT by Inhibition of Hsp70 Chaperone. Int J Mol Sci. 2021;22:6902. 10.3390/ijms22136902 PMC8268552 34199046

[35] Zhang L, Li Z, Fan Y, Li H, Li Z, Li Y (2015). Overexpressed GRP78 affects EMT and cell-matrix adhesion via autocrine TGF-β/Smad2/3 signaling. Int J Biochem Cell Biol.

[36] Ling X, Wan J, Peng B, Chen J (2021). Hsp70 promotes SUMO of HIF-1α and promotes lung cancer invasion and metastasis. J Oncol.

[37] Böckers M, Paul NW, Efferth T (2020). Organophosphate ester tri-o-cresyl phosphate interacts with estrogen receptor α in MCF-7 breast cancer cells promoting cancer growth. Toxicol Appl Pharm.

[38] Tong X, Qu X, Wang M (2021). A four-gene-based prognostic model predicts overall survival in patients with cutaneous melanoma. Front Oncol.

[39] Bonif M, Meuwis MA, Close P, Benoit V, Heyninck K, Chapelle JP (2006). TNFalpha- and IKKbeta-mediated TANK/I-TRAF phosphorylation: implications for interaction with NEMO/IKKgamma and NF-kappaB activation. Biochem J.

[40] Gatot JS, Gioia R, Chau TL, Patrascu F, Warnier M, Close P (2007). Lipopolysaccharide-mediated interferon regulatory factor activation involves TBK1-IKKepsilon-dependent Lys(63)-linked polyubiquitination and phosphorylation of TANK/I-TRAF. J Biol Chem.

[41] Yim EK, Lee KH, Kim CJ, Park JS (2006). Analysis of differential protein expression by cisplatin treatment in cervical carcinoma cells. Int J Gynecol Cancer.

[42] Sanchez-Carbayo M, Belbin TJ, Scotlandi K, Prystowsky M, Baldini N, Childs G (2003). Expression profiling of osteosarcoma cells transfected with MDR1 and NEO genes: regulation of cell adhesion, apoptosis, and tumor suppression-related genes. Lab Invest.

[43] Yang J, Hong Y, Wang W, Wu W, Chi Y, Zong H (2009). HSP70 protects BCL2L12 and BCL2L12A from N-terminal ubiquitination-mediated proteasomal degradation. FEBS Lett.

[44] Shan Y, Gao Y, Zhang L, Ma L, Shi Y, Liu X (2019). H4 receptor inhibits lipopolysaccharide-induced NF-κB activation by interacting with tumor necrosis factor receptor-associated factor 6. Neuroscience.

[45] Lei Z, Xia X, He Q, Luo J, Xiong Y, Wang J (2021). HSP70 promotes tumor progression by stabilizing Skp2 expression in gastric cancer cells. Mol Carcinog.

[46] Xu Y, Yang Q, Xue B, Wang X, Tian R, Deng R (2022). Heat shock-binding protein 21 regulates the innate immune response to viral infection. J Virol.

