# Supplementary material for: Heat shock protein HSPA13 promotes hepatocellular carcinoma progression by stabilizing TANK
Source: Cell Death Discov. 2023 Dec 8;9:443. doi: 10.1038/s41420-023-01735-0 (PMC10703869; doi:10.1038/s41420-023-01735-0)
Supplement: Supplementary file 1 — Supplementary figure legends [file 41420_2023_1735_MOESM1_ESM.docx]

**Supplementary figure 1. HSPA13 promotes HCC cell survival.**

Cell survival of HCC cells with or without HSPA13 knockdown was examined using CCK-8 assay.

**Supplementary figure 2. HSPA13 interacts with TANK.**

1. HitPredict tool was used to predict the proteins that may interact with HSPA13.
2. Co-immunoprecipitation of endogenous HSPA13 and HYOU1 in Huh-7 cells.
3. Co-immunoprecipitation of endogenous HSPA13 and FBX6 in Huh-7 cells.

**Supplementary figure 3. Original images of western blots**
