# Supplementary figures and images for: Heat shock protein HSPA13 promotes hepatocellular carcinoma progression by stabilizing TANK
Source: Cell Death Discov. 2023 Dec 8;9:443. doi: 10.1038/s41420-023-01735-0 (PMC10703869; doi:10.1038/s41420-023-01735-0)

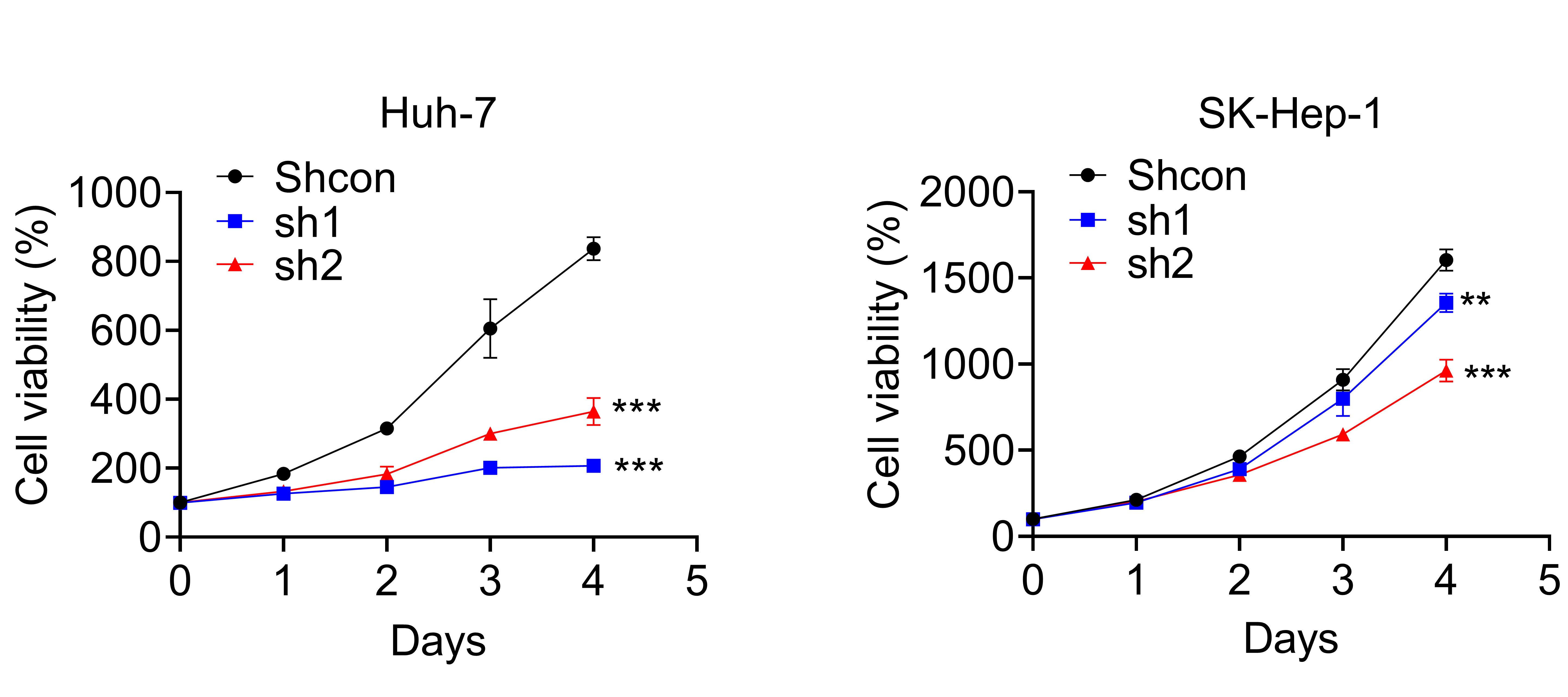

Supplement: Supplementary file 2 — Supplementary figure 1 [file 41420_2023_1735_MOESM2_ESM.tif]

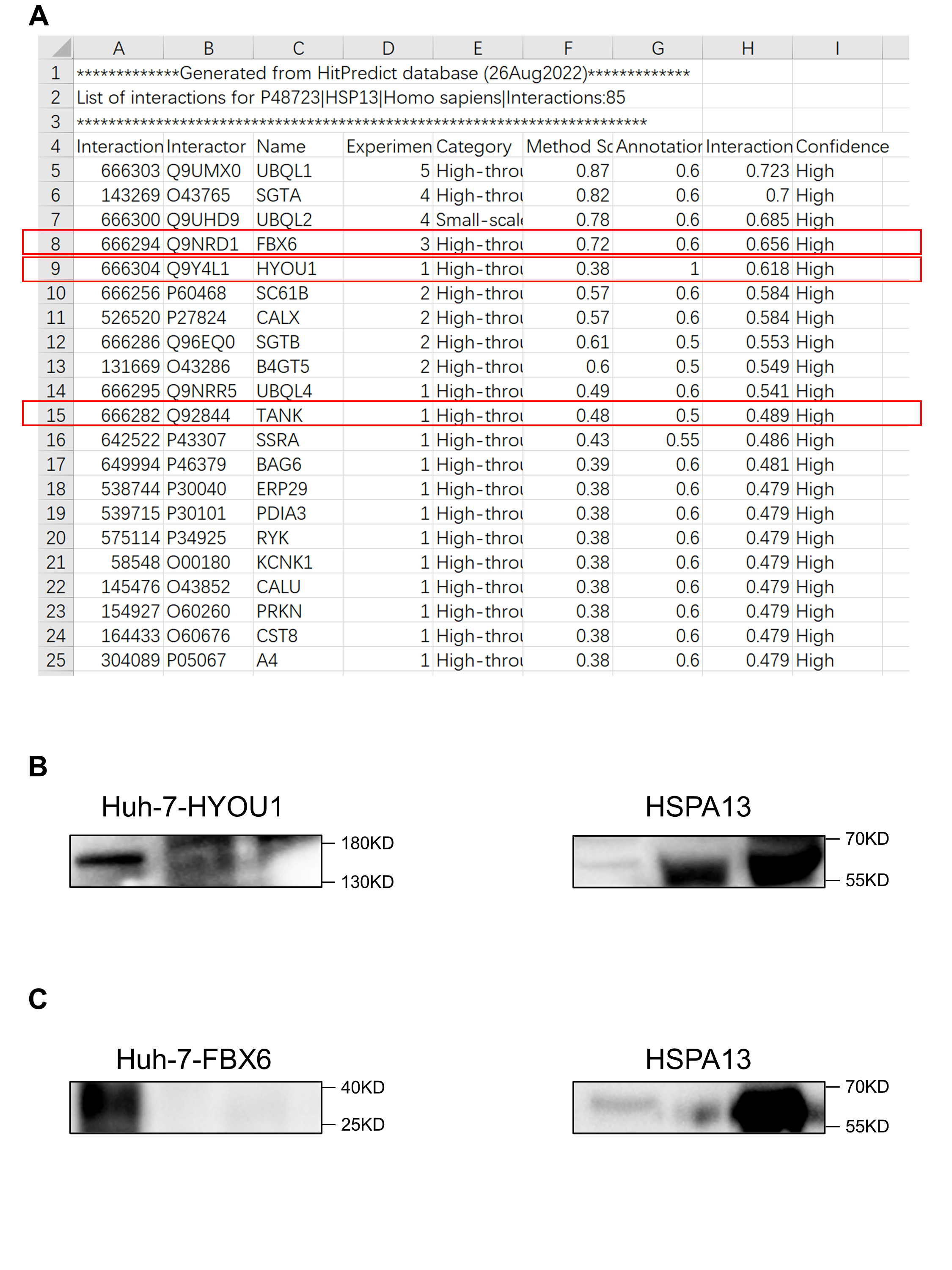

Supplement: Supplementary file 3 — Supplementary figure 2 [file 41420_2023_1735_MOESM3_ESM.tif]
