# Supplementary material for: Heat shock protein HSPA13 promotes hepatocellular carcinoma progression by stabilizing TANK
Source: Cell Death Discov. 2023 Dec 8;9:443. doi: 10.1038/s41420-023-01735-0 (PMC10703869; doi:10.1038/s41420-023-01735-0)

Fig.2A

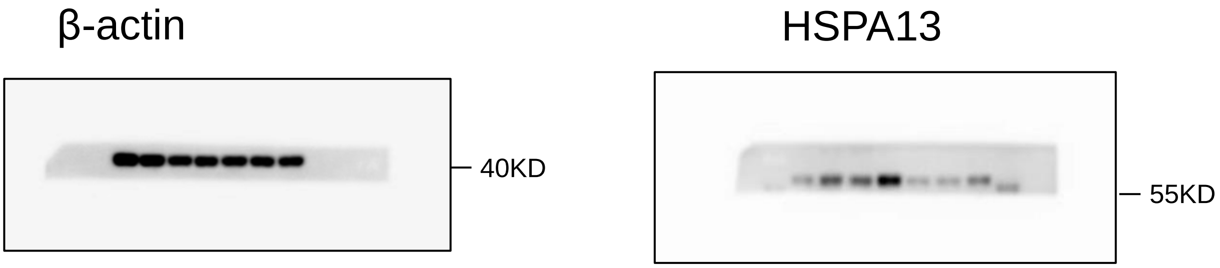
β-actin
40KD
HSPA13
55KD

Fig.2B

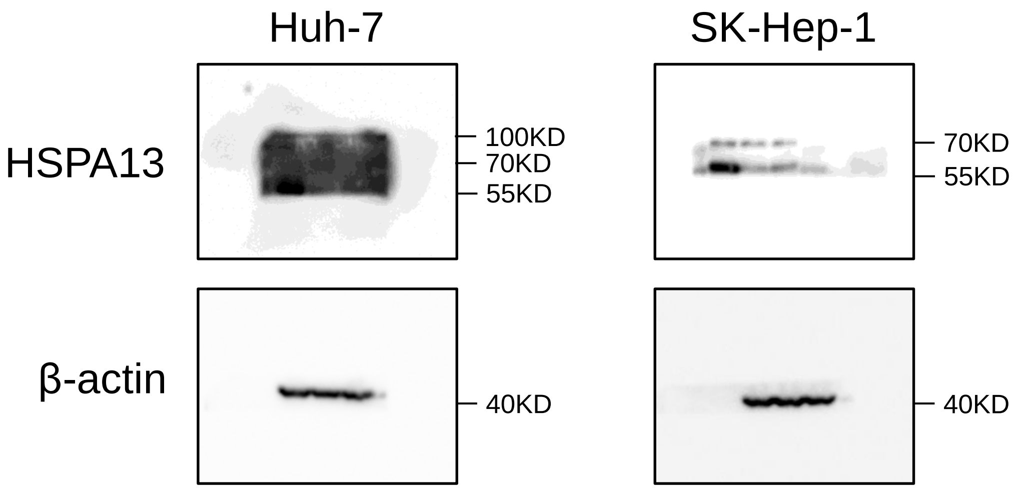
Huh-7
SK-Hep-1
HSPA13
100KD
70KD
55KD
70KD
55KD
β-actin
40KD
40KD

Fig.3A

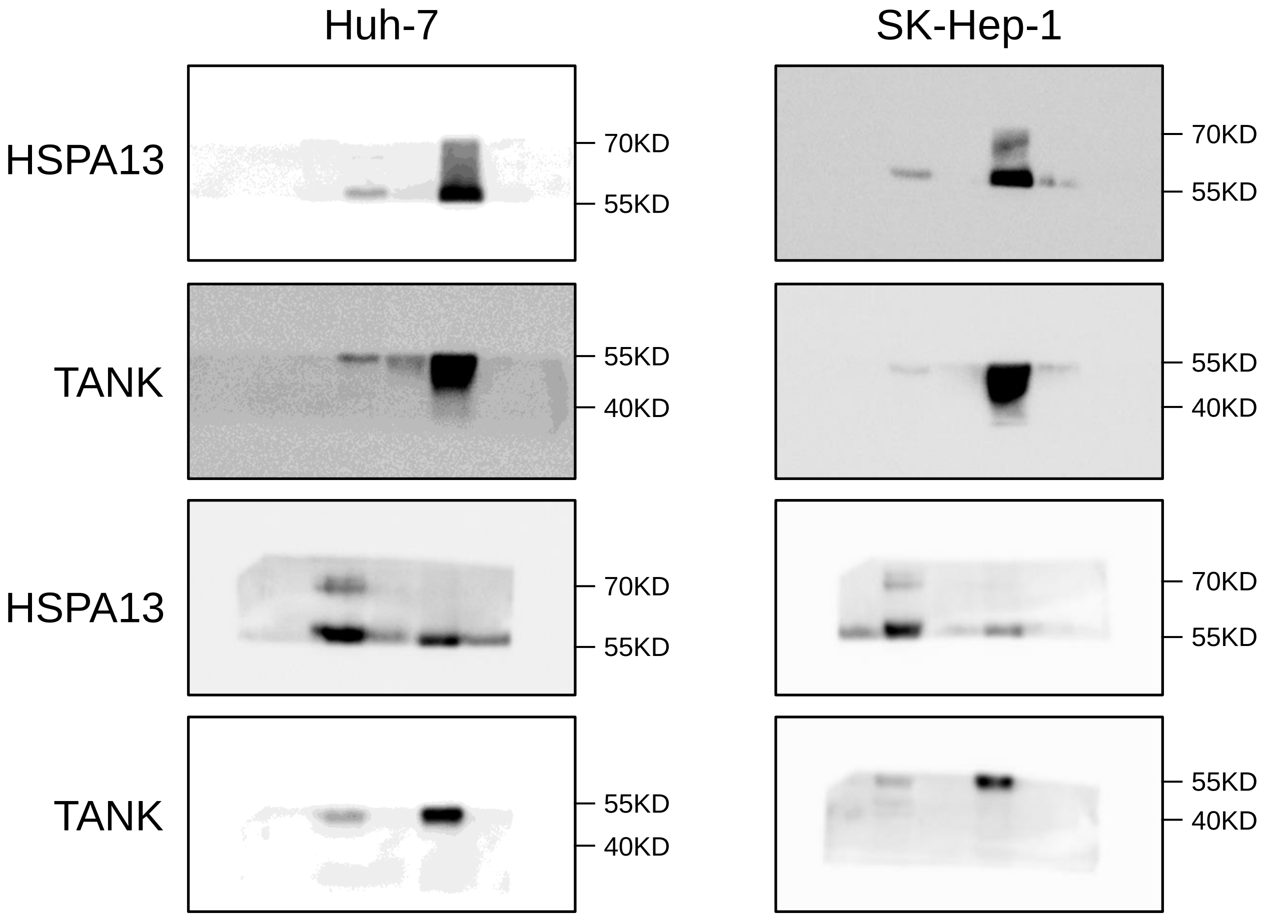
Huh-7
SK-Hep-1
HSPA13
70KD
55KD
70KD
55KD
TANK
55KD
40KD
55KD
40KD
HSPA13
70KD
55KD
70KD
55KD
TANK
55KD
40KD
55KD
40KD

Fig.3B

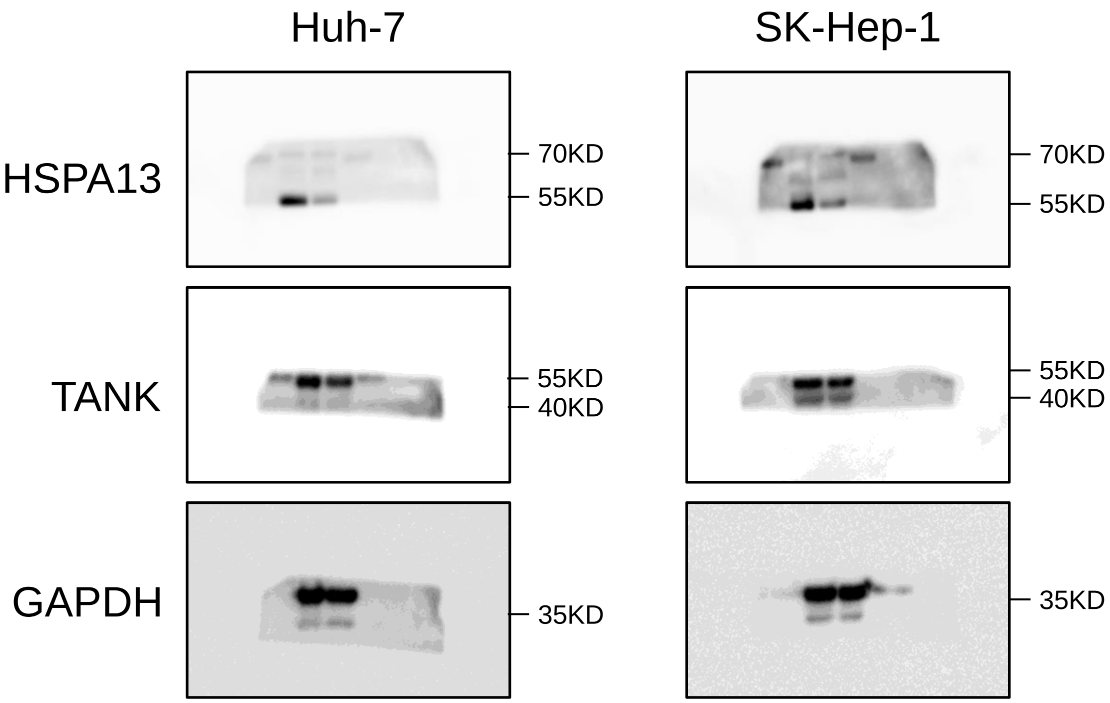
Huh-7
SK-Hep-1
HSPA13
70KD
55KD
70KD
55KD
TANK
55KD
40KD
55KD
40KD
GAPDH
35KD
35KD

Fig.3C

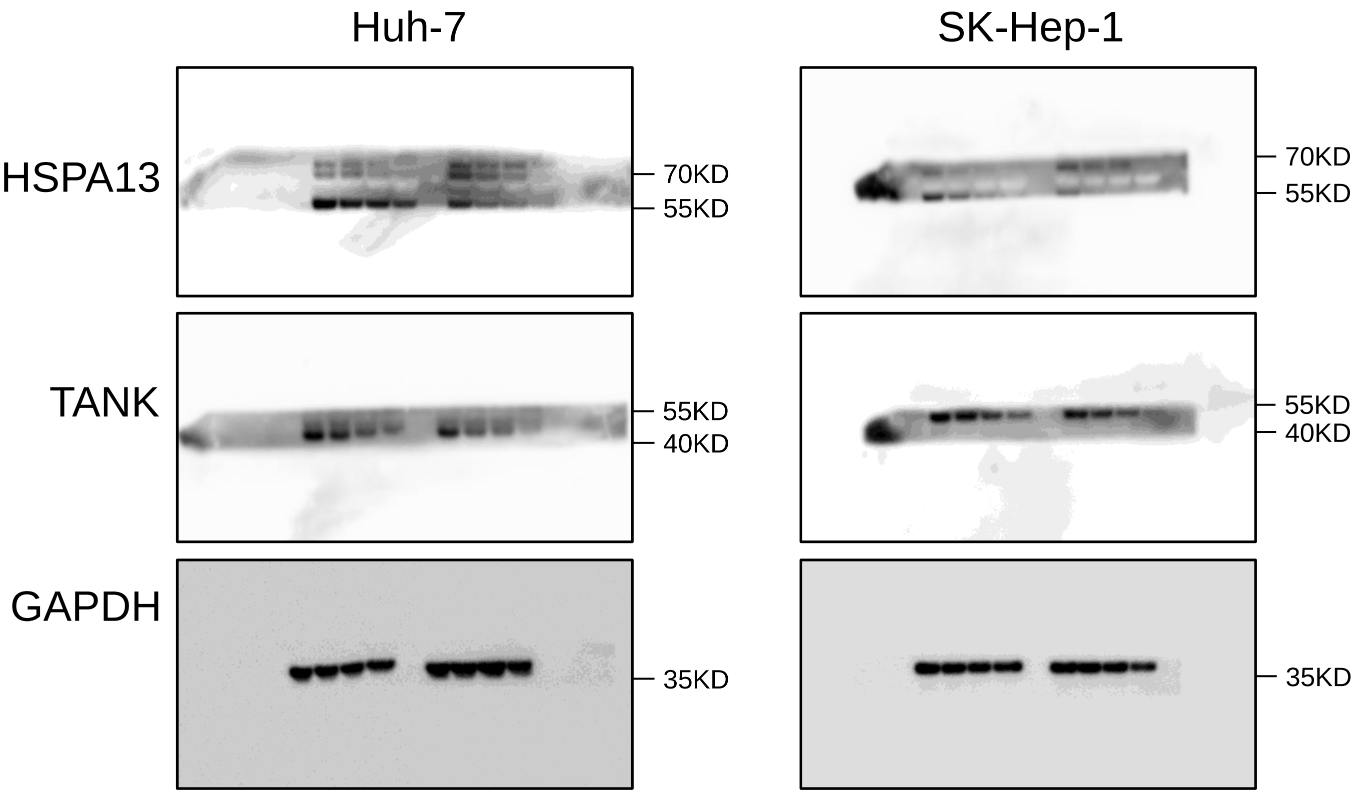
Huh-7
SK-Hep-1
HSPA13
70KD
55KD
70KD
55KD
TANK
55KD
40KD
55KD
40KD
GAPDH
35KD
35KD

Fig.3D

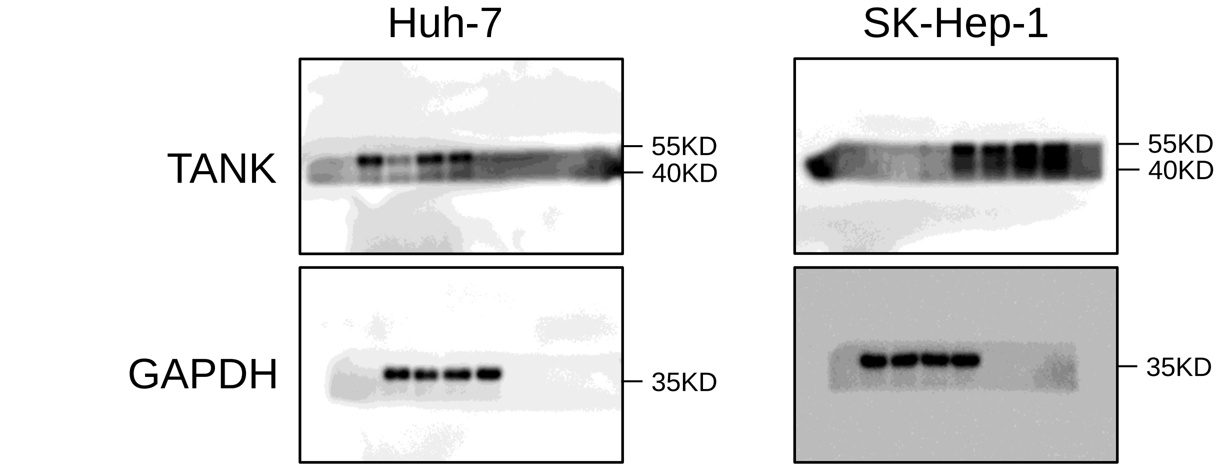
Huh-7
SK-Hep-1
TANK
55KD
40KD
55KD
40KD
GAPDH
35KD
35KD

Fig.3E

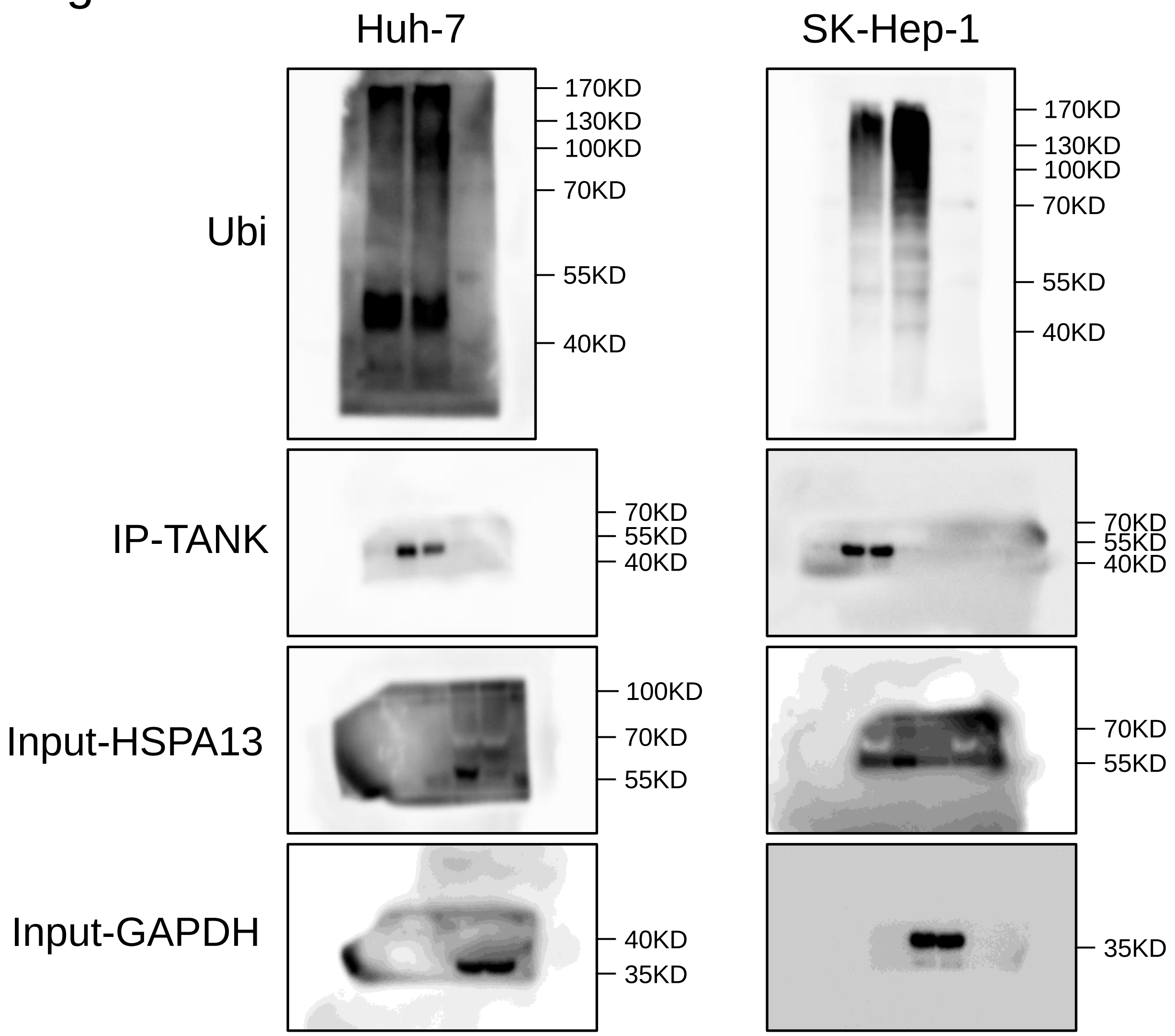
Huh-7
SK-Hep-1
Ubi
170KD
130KD
100KD
70KD
55KD
40KD
170KD
130KD
100KD
70KD
55KD
40KD
IP-TANK
70KD
55KD
40KD
70KD
55KD
40KD
Input-HSPA13
100KD
70KD
55KD
70KD
55KD
Input-GAPDH
40KD
35KD
35KD

Fig.4A

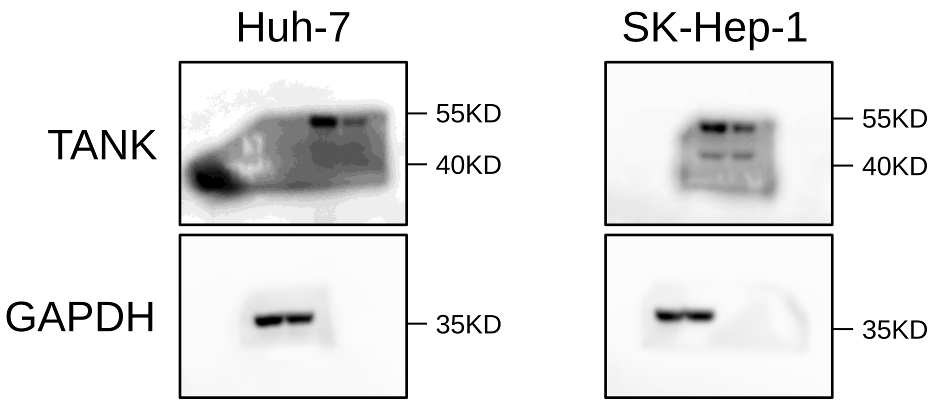
Huh-7
SK-Hep-1
TANK
55KD
40KD
55KD
40KD
GAPDH
35KD
35KD

Fig.5A

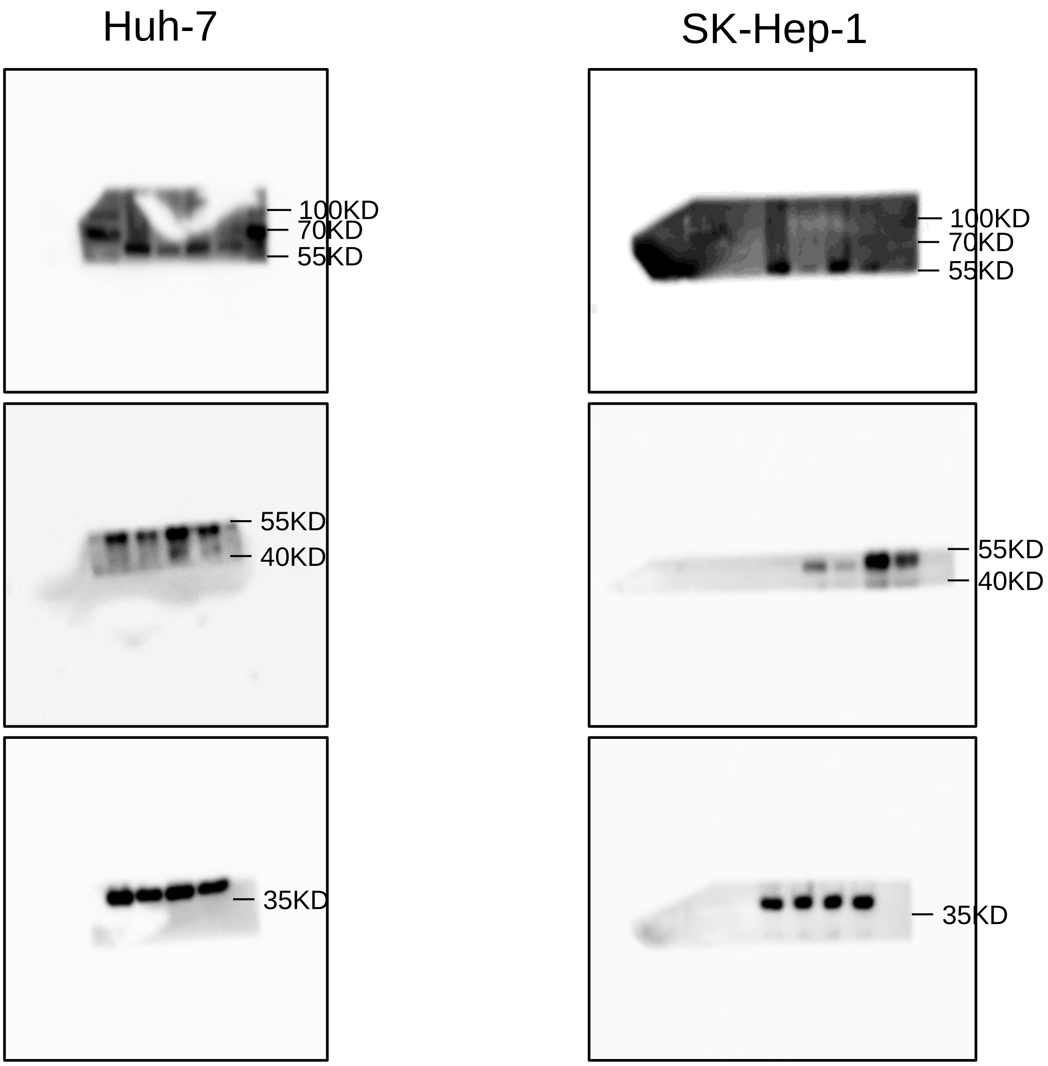
Huh-7
SK-Hep-1
100KD
70KD
55KD
100KD
70KD
55KD
55KD
40KD
55KD
40KD
35KD
35KD

Fig.6A

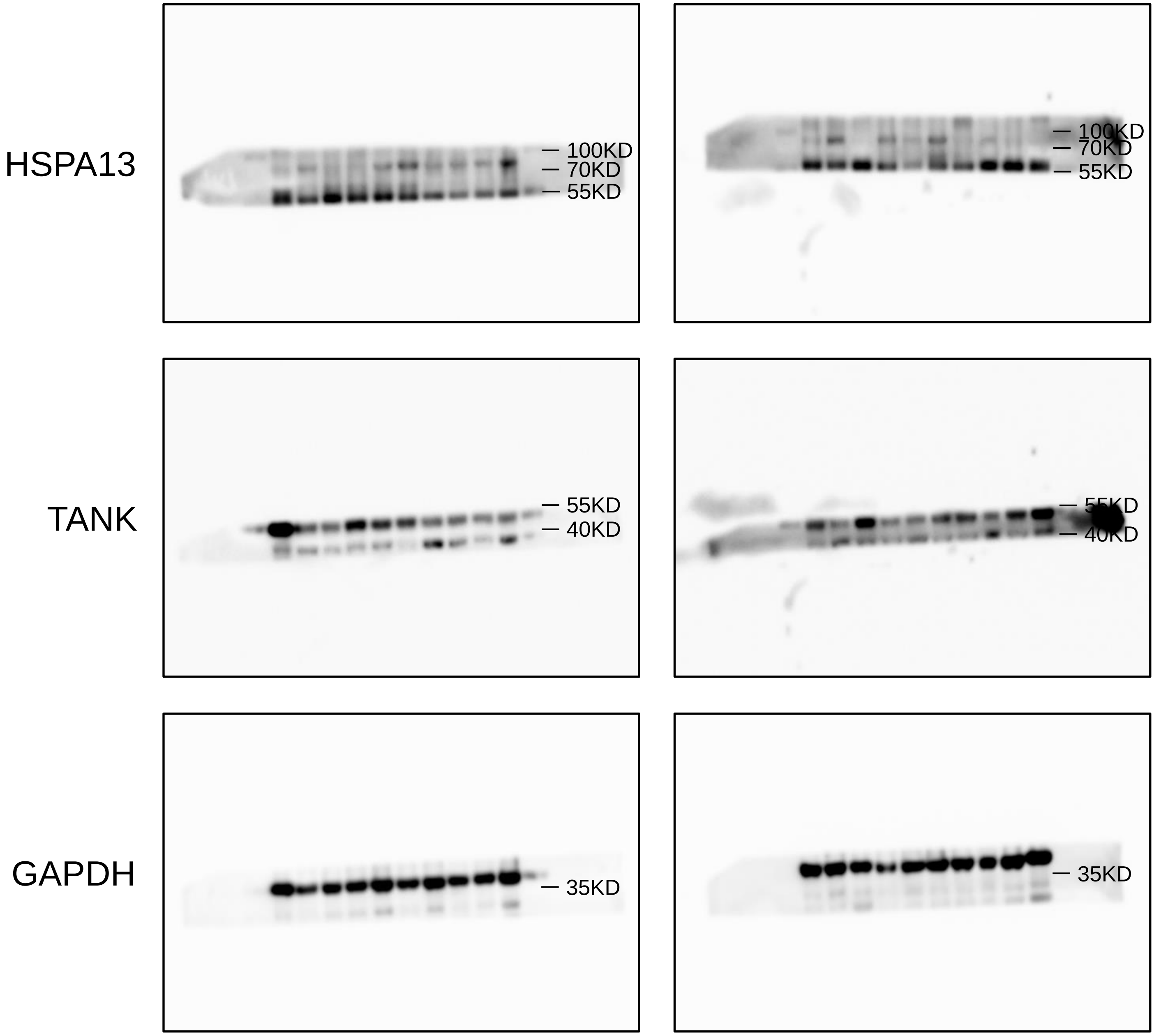
HSPA13
TANK
GAPDH
100KD
70KD
55KD
100KD
70KD
55KD
55KD
40KD
55KD
40KD
35KD
35KD

Supplementary figure 2B

Huh-7-HYOU1

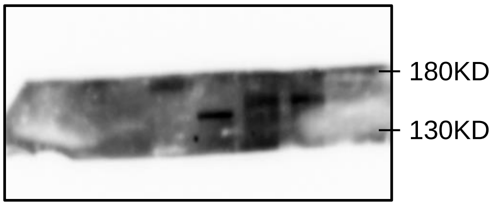

HSPA13

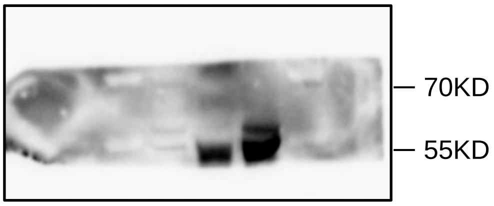

Supplementary figure 2C

Huh-7-FBX6

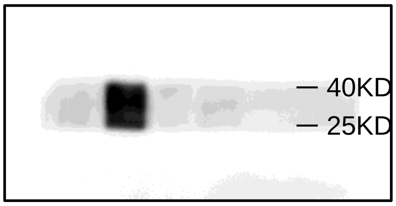

HSPA13

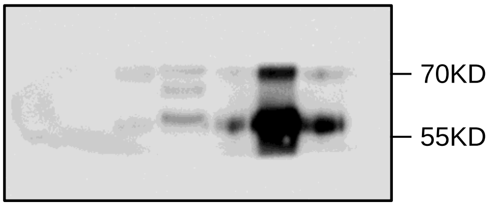

Supplement: Supplementary file 4 — Supplementary figure 3 [file 41420_2023_1735_MOESM4_ESM.pdf]
